# Troponin T, Left Ventricular Ejection Fraction, and Tricuspid Regurgitation Velocity for Biomarker- and Echocardiography-Based Risk Stratification in Critically Ill Patients with Heart Failure

**DOI:** 10.3390/ijms27125339

**Published:** 2026-06-13

**Authors:** Hasan Burak Isleyen, Sevil Tugrul Yavuz, Sercan Bulut, Fatih Kizkapan, Cevahir Alioglu, Ali Arda Sozen, Mahsa Khanmohammadi

**Affiliations:** 1 Department of Cardiology, İstanbul Nişantaşı Üniversitesi, Istanbul 34475, Turkey; 2 Department of Cardiology, İstanbul Başakşehir Çam ve Sakura Şehir Hastanesi, Istanbul 34480, Turkey; seviltugrul@hotmail.com; 3 Department of Cardiology, Bağcılar Eğitim ve Araştırma Hastanesi, Bagcilar 34200, Turkey; srcnbltdr@gmail.com (S.B.); cartilago84@mynet.com (F.K.); 4 Department of Cardiology, Bezmialem Foundation University Medical Faculty Hospital, Istanbul 34093, Turkey; alioglu.ce.86@gmail.com (C.A.); ali.sozen@bezmialem.edu.tr (A.A.S.); mahsa.khanmohammadi@bezmialem.edu.tr (M.K.)

**Keywords:** heart failure, troponin T, left ventricular ejection fraction, tricuspid regurgitation velocity, MIMIC-IV, MIMIC-IV-ECHO, intensive care, Cox regression, risk stratification

## Abstract

Troponin T is a molecular marker of cardiomyocyte injury, whereas left ventricular ejection fraction (LVEF) and tricuspid regurgitation velocity (TRV) reflect downstream ventricular and cardiopulmonary measures. This study evaluated whether synchronized troponin T and echocardiographic data can identify mortality risk in critically ill patients with heart failure, while separating statistical association from clinically meaningful incremental discrimination. Adult intensive care unit admissions with heart failure diagnoses were identified from MIMIC-IV and MIMIC-IV-ECHO. The primary endpoint was 28-day all-cause mortality; one-year mortality was secondary. Multivariable Cox models were adjusted for demographics, comorbidity, illness severity, organ support, and laboratory covariates. Restricted cubic splines, proportional hazards diagnostics, variance inflation factors, prespecified subgroup interaction tests, complete-case analyses, and multiple imputation sensitivity analyses were performed. The final cohort included 4362 patients, and 1072 patients (24.6%) died within 28 days. In the primary complete-case Cox model (*n* = 2087; 659 deaths), higher log-transformed troponin T was associated with higher 28-day mortality (hazard ratio [HR], 1.09; 95% confidence interval [CI], 1.03–1.15; *p* = 0.003), and higher LVEF was associated with lower mortality (HR per percentage point, 0.99; 95% CI, 0.99–1.00; *p* = 0.004). After severity and organ-support covariates were entered, troponin T and LVEF produced statistically detectable but very small C-statistic gains. Measurable TRV was available in 1546 patients and was associated with mortality in that subset (HR, 1.28; 95% CI, 1.08–1.52; *p* = 0.005). Troponin T, LVEF, and TRV were associated with mortality in ICU heart failure. Their contribution was best interpreted as risk enrichment within a clinical severity framework rather than a stand-alone decision rule.

## 1. Introduction

Heart failure remains a major cause of hospital use and death, and intensive care admission selects a group in which myocardial injury, ventricular reserve, pulmonary vascular loading, and multiorgan dysfunction coexist. Contemporary definitions separate heart failure presentations by structural disease, symptoms, and ejection fraction, but acute risk in the intensive care unit (ICU) often depends on the simultaneous burden of cardiac and non-cardiac illness [1,2].

Molecular cardiology has made troponin T a clinically usable marker of cardiomyocyte injury rather than only a diagnostic test for myocardial infarction. Troponin T belongs to the thin-filament troponin complex and is interpreted clinically as a marker of myocardial injury; circulating concentrations may rise with ischemic injury, supply–demand imbalance, membrane disruption, apoptosis, renal dysfunction, or sustained wall stress [3,4].

Left ventricular ejection fraction (LVEF) and tricuspid regurgitation velocity (TRV) capture functional consequences that differ from molecular injury. LVEF represents global left ventricular systolic performance, whereas TRV estimates the pressure gradient between the right ventricle and right atrium and may reflect pulmonary vascular load, left-sided filling pressure, and right-sided stress when interpreted with clinical and echocardiographic context [5,6].

Critically ill patients with heart failure are a clinically important test case for integrated biomarker and imaging interpretation. Troponin T, LVEF, and TRV may each carry prognostic information, but association alone does not establish clinical usefulness. A major unanswered question is whether these variables add decision-relevant information beyond severity scores, organ support, and comorbidity.

The primary objective was to evaluate the association of synchronized troponin T and LVEF with 28-day mortality in critically ill patients with heart failure. Secondary objectives were to quantify incremental model performance after severity and organ-support covariates, evaluate one-year mortality, examine TRV in a complete-case subset, test non-linearity and proportional hazards assumptions, and test whether there was statistical evidence of a troponin T–LVEF interaction.

## 2. Results

The final ICU heart failure cohort included 4362 patients, and 1072 patients (24.6%) died within 28 days. The primary complete-case Cox model included 2087 patients with 659 deaths (31.6%). Measurable TRV was available in 1546 patients, corresponding to 35.4% of the final cohort and 74.1% of the primary complete-case model sample.

**Figure 1:**
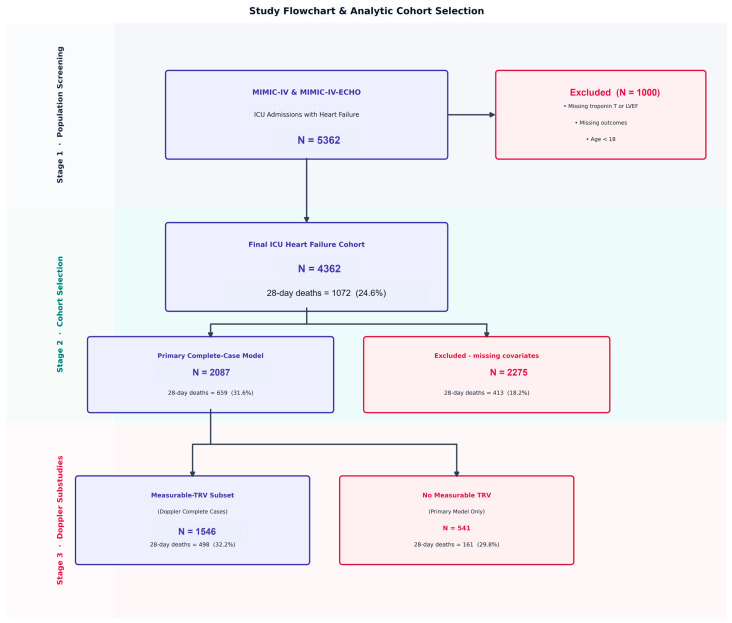
Study flowchart for cohort construction, complete-case modeling, and TRV subset availability. The final analytic cohort included 4362 patients; the primary complete-case Cox model included 2087 patients; the secondary measurable-TRV complete-case model included 1546 patients. TRV, tricuspid regurgitation velocity.

**Figure 2:**
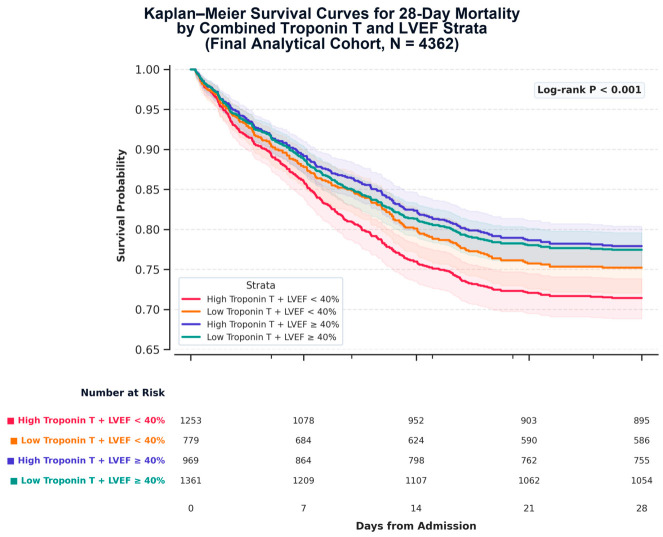
Kaplan–Meier curves for 28-day mortality by combined biomarker–left ventricular ejection fraction (LVEF) strata. Shaded areas indicate 95% confidence intervals.

**Figure 3:**
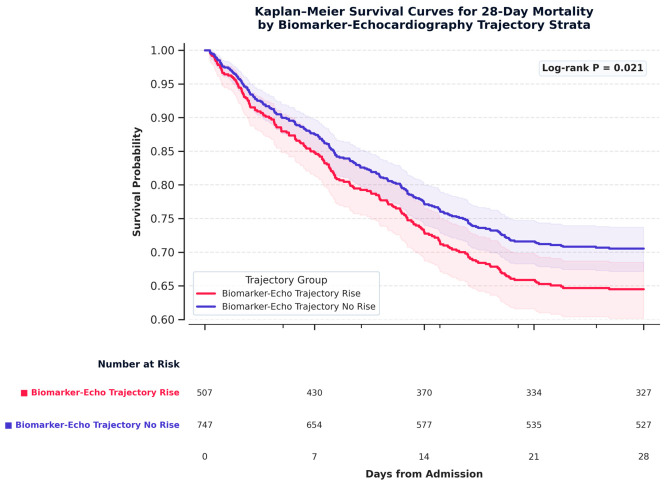
Kaplan–Meier curves for 28-day mortality by biomarker–echocardiography trajectory strata. Shaded areas indicate 95% confidence intervals.

**Figure 4:**
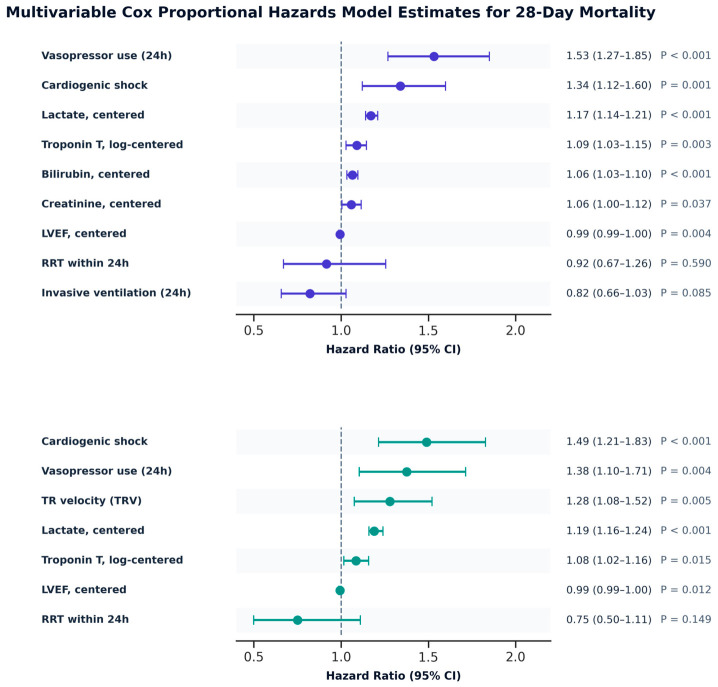
Forest plot summarizing the primary complete-case Cox model and the secondary TRV model.

**Figure 5:**
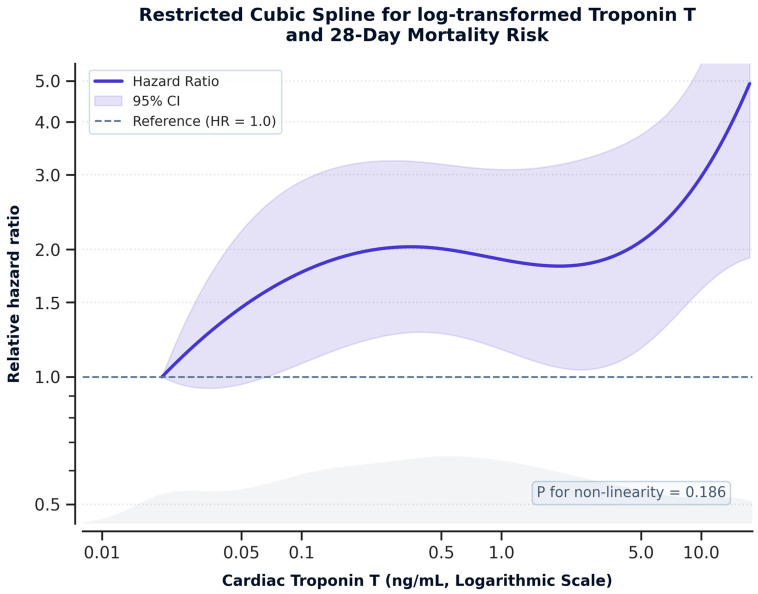
Restricted cubic spline analysis for log-transformed troponin T and 28-day mortality. The revised plot uses the corrected data and is interpreted as a continuous association rather than a threshold model.

**Figure 6:**
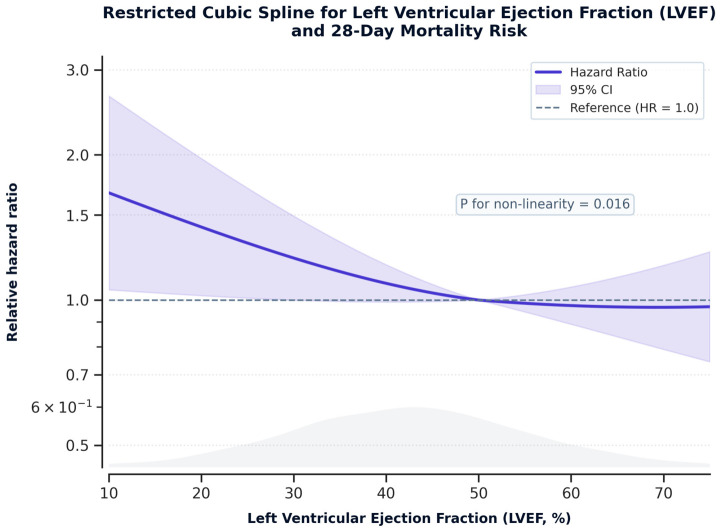
Restricted cubic spline analysis for left ventricular ejection fraction (LVEF) and 28-day mortality. Shaded areas indicate 95% confidence intervals. The non-linearity test was significant, so the curve is interpreted descriptively.

The cohort flow is shown in Figure 1. Kaplan–Meier curves are shown in Figure 2 and Figure 3. The forest plot summarizes the primary complete-case Cox model and the secondary TRV model in Figure 4. Analytic cohorts and endpoint counts are summarized in Table 1. Key multivariable Cox estimates are shown in Table 2. Sequential discrimination is summarized in Table 3. The restricted cubic spline analyses for log-transformed troponin T and LVEF are shown in Figure 5 and Figure 6. The secondary TRV model is shown in Table 4. Full model estimates, sensitivity analyses, diagnostic checks, subgroup interaction tests, and the TRV availability audit are provided in Tables S1–S5. Subgroup forest plots, endpoint comparison, incremental-discrimination plots, and the TRV availability audit are provided in Figures S1–S5.

In the primary complete-case model, log-transformed troponin T was associated with higher 28-day mortality (HR, 1.09; 95% CI, 1.03–1.15; *p* = 0.003). LVEF was associated with lower mortality (HR per percentage point, 0.99; 95% CI, 0.99–1.00; *p* = 0.004). The direction of association was preserved at one year for troponin T (HR, 1.05; 95% CI, 1.01–1.10; *p* = 0.026) and LVEF (HR, 0.99; 95% CI, 0.99–1.00; *p* < 0.001).

Incremental discrimination was modest after severity and organ-support variables were included. The model C-statistic increased from 0.7565 after organ support to 0.7574 after troponin T was added and to 0.7575 after LVEF was added. The troponin T–LVEF interaction raised the C-statistic to 0.7577 and did not materially improve model fit (likelihood ratio *p* = 0.226).

TRV analysis was confined to the measurable-TRV subset. TRV was associated with higher 28-day mortality in the measurable-TRV subset (HR, 1.28; 95% CI, 1.08–1.52; *p* = 0.005). Because patients with measurable TRV may differ systematically from those without measurable TRV, this finding is presented as secondary and not as evidence that TRV should be generalized to the full cohort.

Spline analyses did not support a non-linear association for log-transformed troponin T (non-linearity *p* = 0.186). LVEF showed evidence of non-linearity (non-linearity *p* = 0.016), with the risk curve supporting weaker discrimination at higher LVEF ranges. Variance inflation factors remained below the prespecified threshold (maximum VIF, 2.15).

Approximate proportional hazards diagnostics did not detect significant residual-time correlation for log-transformed troponin T (*p* = 0.165) or LVEF (*p* = 0.541). The interaction term showed possible time-varying behavior in the approximate diagnostic (*p* = 0.019), reinforcing the decision to avoid a mechanistic interpretation of the non-significant interaction.

Multiple imputation sensitivity analyses were consistent with complete-case estimates. Log-transformed troponin T remained associated with 28-day mortality (HR, 1.12; 95% CI, 1.02–1.23; *p* = 0.019), and LVEF remained inversely associated with 28-day mortality (HR, 0.99; 95% CI, 0.99–1.00; *p* = 0.030). Prespecified subgroup analyses did not identify a stable interaction pattern that changed the primary interpretation.

## 3. Discussion

This study evaluated a molecular injury biomarker and echocardiographic measures within the same ICU heart failure framework. Troponin T, LVEF, and TRV were associated with mortality, but the incremental discrimination of troponin T and LVEF beyond severity and organ-support covariates was small. This distinction changes the interpretation of the work: the findings support risk enrichment and pathophysiologic framing rather than a deployable bedside decision rule.

Troponin T represents cardiomyocyte injury at the biomarker level. Aimo et al. reported that high-sensitivity troponin T predicted mortality and heart failure hospitalization in chronic heart failure, and Kociol et al. summarized mechanisms that include myocyte injury, wall stress, renal dysfunction, and neurohormonal activation [7,8]. The present ICU cohort extends this signal into a sicker population, but the estimate cannot isolate ischemic necrosis from critical illness injury, renal dysfunction, or assay heterogeneity.

Acute heart failure studies have also shown that serial troponin T measurements can reflect risk during decompensation [9]. The present analysis is consistent with that literature, but it should not be read as a threshold study or as evidence that a single troponin value should determine treatment intensity.

The LVEF finding is clinically plausible but not sufficient for mechanistic stratification by itself. Three-dimensional and quantitative echocardiographic recommendations illustrate how measurement technique can influence systolic indices [10]. Because MIMIC-IV-ECHO provides structured numeric LVEF without a uniform method field for all examinations, the association should be read as the prognostic value of clinically reported LVEF rather than the effect of a single standardized measurement protocol.

The TRV result adds a cardiopulmonary dimension but carries the strongest selection concern. Pulmonary hypertension guidance emphasizes that TRV should be interpreted with a full right-heart assessment rather than in isolation [11]. Cocianni et al. linked tricuspid regurgitation-related measures with outcomes in acute heart failure, which aligns with the direction of the present TRV estimate [12]. In this study, measurable TRV was available in only 35.4% of the final cohort, so the TRV result is deliberately presented as a secondary complete-case finding.

The revision also addresses clinical incremental value. Added-marker evaluation requires more than a statistically significant coefficient because even strong associations may add little discrimination after established severity variables are included [13,14]. In this cohort, the C-statistic gains after troponin T and LVEF were less than 0.001 each after organ support was entered. That result argues against any claim that these variables alone meaningfully change clinical decision-making in the ICU.

The non-significant troponin T–LVEF interaction limits biological interpretation. The two variables may represent different clinical domains of myocardial injury and systolic function, but the model did not show that their product term improved fit or discrimination. The discussion was therefore revised to remove language that implied a direct biological interaction beyond the data. Clinical utility would require calibration, external validation, and decision-curve thresholds before implementation [15,16].

Several limitations remain. Retrospective coding may produce a heterogeneous heart failure cohort. Troponin T assay generation and analyzer information were not uniformly available, and measurement heterogeneity may have diluted true associations or limited threshold interpretation. The LVEF method was not consistently encoded. TRV availability was incomplete and may reflect selection by image quality or clinical suspicion. External validation was not performed, so transportability to non-MIMIC ICU systems is unknown. The study was not designed as a treatment-response or decision-impact analysis, and reporting does not convert the models into a deployable clinical prediction tool [17].

The next step is an externally validated ICU heart failure model that prespecifies troponin assay handling, standardized LVEF extraction, TRV availability modeling, calibration, and decision-curve thresholds before these variables are used to guide clinical actions.

## 4. Materials and Methods

This retrospective cohort study used de-identified critical care and echocardiography data. Reporting followed STROBE and RECORD guidance for observational studies that use routinely collected health data [18,19].

Data were obtained from MIMIC-IV and MIMIC-IV-ECHO through PhysioNet. MIMIC-IV contains de-identified hospital and ICU data from Beth Israel Deaconess Medical Center [20,21]. MIMIC-IV-ECHO provides structured echocardiographic measurements linked to MIMIC-IV patients [22].

Eligible admissions were adult ICU stays with heart failure diagnosis codes and available outcome follow-ups. The analytic exposure window required troponin T and numeric LVEF to be available within the prespecified clinical window used for the extraction. When multiple values were available, the temporally aligned value closest to the index echocardiogram was retained. The final cohort included 4362 patients; the primary complete-case Cox model included 2087 patients with complete covariate data, troponin T, and LVEF.

Troponin T was treated as a continuous molecular injury biomarker after log transformation and centering. This choice reduced the influence of right-skewed values and avoided dependence on a single assay threshold. The source data identify cardiac troponin T measurements but do not uniformly encode assay platform, generation, or analyzer. Potential heterogeneity in troponin T testing across the study period was therefore handled as a measurement limitation rather than a basis for fixed threshold inference.

LVEF was extracted from the structured numeric percentage field in MIMIC-IV-ECHO. The source does not consistently distinguish visual estimation, Simpson biplane quantification, or alternative measurement techniques across all reports. The analysis therefore treated LVEF as the clinically reported numeric percentage rather than as a method-specific imaging endpoint.

TRV was analyzed as a prespecified secondary echocardiographic variable because a measurable TRV requires an adequate Doppler signal and was unavailable in a substantial proportion of patients. The TRV model was restricted to complete cases with measurable TRV and was not treated as representative of all ICU heart failure patients.

The primary endpoint was 28-day all-cause mortality. One-year all-cause mortality was analyzed as a secondary endpoint. Patients were followed from the index ICU time point used in cohort construction, and survival time was censored at the endpoint-specific horizon.

Cox proportional hazards models were built sequentially. The base model included demographic and comorbidity variables. Subsequent models added illness severity, organ support, troponin T, LVEF, and then the troponin T–LVEF interaction. Multivariable models included clinically selected covariates rather than univariable screening. Harrell C, likelihood ratio tests, and changes in C-statistic were used to separate statistical association from incremental discrimination [23,24].

Restricted cubic splines tested non-linearity for troponin T and LVEF. Approximate proportional hazards diagnostics used residual-time correlations for the primary exposure terms and the interaction term. Variance inflation factors assessed collinearity. Prespecified subgroup analyses tested interaction terms across clinically relevant strata. Multiple imputation with five imputed datasets was used as a sensitivity analysis for missing covariate values [25,26].

No laboratory instruments or reagents were used because this was a retrospective database study. Data extraction was performed using Google BigQuery (Google LLC, Mountain View, CA, USA; accessed 26 April 2026). Statistical analyses were performed using Python version 3.13.5 (Python Software Foundation, Wilmington, DE, USA; accessed 10 June 2026). Continuous variables are summarized as medians with interquartile ranges or means with standard deviations according to distribution in the source tables. Effect estimates are reported as hazard ratios (HRs) with 95% confidence intervals (CIs). A two-sided p value below 0.05 was treated as statistically significant, with emphasis placed on effect size and consistency across analyses.

## 5. Conclusions

Troponin T, LVEF, and TRV were associated with mortality in critically ill patients with heart failure. After severity and organ-support covariates were included, troponin T and LVEF added only small discrimination gains. The final interpretation supports biomarker-echocardiography risk enrichment rather than stand-alone clinical decision-making or mechanistic proof of a biological interaction.

## Figures and Tables

**Table 1:** Analytic cohorts, TRV availability, and endpoint counts.

Analysis Set	*n*	28-Day Deaths	Proportion	Interpretation
Final ICU HF cohort	4362	1072 (24.6%)	100.0%	All eligible patients with heart failure-coded ICU admissions and 28-day outcome data.
Primary complete-case model	2087	659 (31.6%)	47.8%	Patients with troponin T, numeric LVEF, and complete adjustment covariates.
Measurable-TRV subset	1546	498 (32.2%)	35.4%	Secondary complete-case subset with TRV available.
No measurable TRV in final cohort	2816	574 (20.4%)	64.6%	Patients excluded from TRV-specific modeling.
No measurable TRV within primary complete-case model	541	161 (29.8%)	25.9% of primary complete-case sample	Patients included in primary model but not in TRV subset.

HF, heart failure; ICU, intensive care unit; LVEF, left ventricular ejection fraction; TRV, tricuspid regurgitation velocity.

**Table 2:** Key multivariable Cox model estimates.

Variable	28-Day HR (95% CI)	*p*	1-Year HR (95% CI)	*p*
Troponin T, log-transformed and centered	1.09 (1.03–1.15)	0.003	1.05 (1.01–1.10)	0.026
LVEF, centered percentage points	0.99 (0.99–1.00)	0.004	0.99 (0.99–1.00)	<0.001
Cardiogenic shock	1.34 (1.12–1.60)	0.001	1.19 (1.03–1.38)	0.017
Vasopressor use within 24 h	1.53 (1.27–1.85)	<0.001	1.47 (1.27–1.70)	<0.001
Invasive ventilation within 24 h	0.82 (0.66–1.03)	0.085	0.86 (0.72–1.03)	0.094
Renal replacement therapy within 24 h	0.92 (0.67–1.26)	0.590	1.15 (0.89–1.49)	0.280
Creatinine, centered	1.06 (1.00–1.12)	0.037	1.07 (1.02–1.11)	0.002
Lactate, centered	1.17 (1.14–1.21)	<0.001	1.13 (1.11–1.16)	<0.001
Bilirubin, centered	1.06 (1.03–1.10)	<0.001	1.06 (1.03–1.08)	<0.001

Troponin T was log-transformed and centered. LVEF is reported per percentage point. Full model variables are provided in Table S1.

**Table 3:** Incremental discrimination across sequential 28-day mortality models.

Sequential Model	*n*	Events	Harrell C	ΔC vs. Previous	ΔC vs. M1	LRT *p*
Demographics and comorbidities	2087	659	0.6443		+0.0000	
Plus illness severity	2087	659	0.7535	+0.1093	+0.1093	
Plus organ support	2087	659	0.7565	+0.0029	+0.1122	
Plus troponin T	2087	659	0.7574	+0.0010	+0.1132	<0.001
Plus LVEF	2087	659	0.7575	+0.0001	+0.1132	0.023
Plus troponin T × LVEF	2087	659	0.7577	+0.0002	+0.1134	0.226

The C-statistic gains after troponin T and LVEF were statistically detectable but small after severity and organ-support variables were included.

**Table 4:** Secondary Complete-Case TRV Model.

Variable	28-Day HR (95% CI)	*p*
Troponin T, log-transformed	1.08 (1.02–1.16)	0.015
LVEF	0.99 (0.99–1.00)	0.012
TRV	1.28 (1.08–1.52)	0.005
Cardiogenic shock	1.49 (1.21–1.83)	<0.001
Lactate	1.19 (1.16–1.24)	<0.001
Vasopressor use within 24 h	1.38 (1.10–1.71)	0.004
Renal replacement therapy within 24 h	0.75 (0.50–1.11)	0.149

The TRV model was restricted to patients with measurable TRV and is interpreted as secondary because of incomplete TRV availability.

## Data Availability

MIMIC-IV v3.1 and MIMIC-IV-ECHO v1.0 are available through PhysioNet to credentialed users under the applicable data use agreements. Derived code lists, analysis-ready variable definitions, and supplementary statistical summaries are provided in the Supplementary Materials.

## Supplementary Materials

The following supporting information can be downloaded at: https://www.mdpi.com/article/10.3390/ijms27125339/s1.

## Abbreviations

CI, confidence interval; HF, heart failure; HR, hazard ratio; ICU, intensive care unit; LVEF, left ventricular ejection fraction; MICE, multiple imputation by chained equations; PH, proportional hazards; RECORD, Reporting of Studies Conducted Using Observational Routinely Collected Health Data; RCS, restricted cubic spline; STROBE, Strengthening the Reporting of Observational Studies in Epidemiology; TRV, tricuspid regurgitation velocity; VIF, variance inflation factor.
