# Supplementary material for: Troponin T, Left Ventricular Ejection Fraction, and Tricuspid Regurgitation Velocity for Biomarker- and Echocardiography-Based Risk Stratification in Critically Ill Patients with Heart Failure
Source: Int J Mol Sci. 2026 Jun 13;27(12):5339. doi: 10.3390/ijms27125339 (PMC13299282; doi:10.3390/ijms27125339)
Supplement: Supplementary file 1 [file ijms-27-05339-s001.zip › Additional_File_S1_STROBE_RECORD_Checklist_revised.pdf]

Additional File S1. STROBE/RECORD Checklist

STROBE/RECORD mapping for the revised manuscript.

Checklist domain	Location	Revision note
Title/abstract	Addressed in title and abstract	Study design, data sources, exposure variables, endpoints, and conservative conclusion are stated.
Introduction/background	Addressed in Introduction	Troponin T molecular biomarker context, LVEF, TRV, and ICU HF rationale are described.
Objectives	Addressed in Introduction	Primary, secondary, incremental-value, and diagnostic objectives are specified.
Study design	Addressed in Methods	Retrospective cohort design using MIMIC-IV and MIMIC-IV-ECHO is stated.
Setting/data sources	Addressed in Methods and Data Availability	PhysioNet access and de-identified data source are stated.
Participants	Addressed in Methods and Figure 1	Eligibility, complete-case model sample, and TRV subset are shown.
Variables	Addressed in Methods	Troponin T, LVEF, TRV, covariates, and endpoints are defined.
Bias	Addressed in Methods and Discussion	Selection bias from TRV availability and retrospective measurement are explicitly discussed.
Study size	Addressed in Results and Table 1	Final cohort, primary complete-case model, and TRV subset counts are reported.
Statistical methods	Addressed in Methods and Supplement	Cox models, splines, PH diagnostics, VIF, MICE, subgroup tests, and model performance are described.
Results	Addressed in Results and Tables	Primary estimates, model performance, TRV subset, and sensitivity checks are reported.
Limitations	Addressed in Discussion	HF heterogeneity, assay heterogeneity, LVEF extraction method, TRV selection, missing data, and external validation are discussed.
Interpretation	Addressed in Discussion and Conclusions	Claims are tempered; association is separated from clinical decision-making value.
Funding	Addressed in Declarations	No specific funding reported.
