# Supplementary material for: Troponin T, Left Ventricular Ejection Fraction, and Tricuspid Regurgitation Velocity for Biomarker- and Echocardiography-Based Risk Stratification in Critically Ill Patients with Heart Failure
Source: Int J Mol Sci. 2026 Jun 13;27(12):5339. doi: 10.3390/ijms27125339 (PMC13299282; doi:10.3390/ijms27125339)
Supplement: Supplementary file 1 [file ijms-27-05339-s001.zip › Additional_File_S2_Supplementary_Methods_and_Results_revised.pdf]

Additional File S2. Supplementary Methods and Results

Supplementary Methods

The revised analysis plan kept the original cohort and endpoints unchanged. Additional revision analyses were targeted to reviewer comments and did not alter the primary exposure definitions.

Troponin T was retained as a log-transformed continuous biomarker. Assay generation and analyzer information were not uniformly encoded in the extracted source fields. Therefore, the revision avoids fixed diagnostic thresholds and treats potential assay heterogeneity as a limitation.

LVEF was extracted from the structured numeric percentage field in MIMIC-IV-ECHO. Because the source does not consistently encode whether values were visually estimated, Simpson biplane-derived, or obtained by another technique, method-specific LVEF analyses were not performed.

TRV analysis was prespecified as secondary complete-case modeling. The revision adds an availability audit because measurable TRV depends on Doppler signal quality, report completeness, and clinical acquisition patterns.

Model diagnostics included restricted cubic spline non-linearity tests, variance inflation factors, approximate proportional hazards checks, sequential Harrell C-statistics, likelihood ratio tests, and multiple imputation sensitivity analyses.

Supplementary Results

The final cohort included 4362 patients with 1072 28-day deaths. The primary complete-case analysis included 2087 patients with 659 deaths. Measurable TRV was available in 1546 patients.

After organ-support covariates were included, the Harrell C-statistic was 0.7565. Adding troponin T increased the C-statistic to 0.7574, and adding LVEF increased it to 0.7575. The troponin T–LVEF interaction increased the C-statistic to 0.7577 and had a likelihood ratio P value of 0.226.

The non-linearity test was not significant for log-transformed troponin T (P = 0.186) and was significant for LVEF (P = 0.016). The maximum VIF was 2.15.

Approximate proportional hazards diagnostics did not identify significant time-dependence for log-transformed troponin T or LVEF; the interaction term had a significant diagnostic result and was therefore interpreted conservatively.

MICE sensitivity analyses supported the same direction of effect for troponin T and LVEF as the complete-case models.
