# Supplementary material for: Troponin T, Left Ventricular Ejection Fraction, and Tricuspid Regurgitation Velocity for Biomarker- and Echocardiography-Based Risk Stratification in Critically Ill Patients with Heart Failure
Source: Int J Mol Sci. 2026 Jun 13;27(12):5339. doi: 10.3390/ijms27125339 (PMC13299282; doi:10.3390/ijms27125339)
Supplement: Supplementary file 1 [file ijms-27-05339-s001.zip › Additional_File_S3_Supplementary_Tables_revised.pdf]

Additional File S3. Supplementary Tables

Supplementary Table S1. Full primary multivariable Cox model estimates.

Variable	28-day HR (95% CI)	P	1-year HR (95% CI)	P
Cardiogenic shock	1.34 (1.12–1.60)	0.001	1.19 (1.03–1.38)	0.017
Creatinine, centered	1.06 (1.00–1.12)	0.037	1.07 (1.02–1.11)	0.002
Lactate, centered	1.17 (1.14–1.21)	<0.001	1.13 (1.11–1.16)	<0.001
Bilirubin, centered	1.06 (1.03–1.10)	<0.001	1.06 (1.03–1.08)	<0.001
Invasive ventilation within 24 h	0.82 (0.66–1.03)	0.085	0.86 (0.72–1.03)	0.094
Vasopressor use within 24 h	1.53 (1.27–1.85)	<0.001	1.47 (1.27–1.70)	<0.001
Renal replacement therapy within 24 h	0.92 (0.67–1.26)	0.590	1.15 (0.89–1.49)	0.280
Troponin T, log-transformed and centered	1.09 (1.03–1.15)	0.003	1.05 (1.01–1.10)	0.026
LVEF, centered percentage points	0.99 (0.99–1.00)	0.004	0.99 (0.99–1.00)	<0.001

Variables are shown as modeled in the complete-case primary analysis.

Supplementary Table S2. Sensitivity analyses.

Sensitivity cohort	Variable	N	Events	HR (95% CI)	P
After ICU only	Troponin T, log-transformed and centered	1916	603	1.08 (1.02–1.15)	0.005
After ICU only	LVEF, centered percentage points	1916	603	0.99 (0.99–1.00)	0.006
Echo within 24h	Troponin T, log-transformed and centered	1696	512	1.09 (1.02–1.16)	0.007
Echo within 24h	LVEF, centered percentage points	1696	512	0.99 (0.99–1.00)	0.021
Echo within 48h	Troponin T, log-transformed and centered	1849	567	1.08 (1.02–1.14)	0.013
Echo within 48h	LVEF, centered percentage points	1849	567	0.99 (0.99–1.00)	0.005
No acute MI	Troponin T, log-transformed and centered	1131	366	1.15 (1.06–1.24)	<0.001
No acute MI	LVEF, centered percentage points	1131	366	1.00 (0.99–1.00)	0.245
No cardiogenic shock	Troponin T, log-transformed and centered	1407	383	1.10 (1.02–1.19)	0.014
No cardiogenic shock	LVEF, centered percentage points	1407	383	0.99 (0.99–1.00)	0.122
TRV subset	Troponin T, log-transformed and centered	1546	498	1.08 (1.01–1.15)	0.017
TRV subset	LVEF, centered percentage points	1546	498	0.99 (0.99–1.00)	0.003
TRV subset	TRV	1546	498	1.28 (1.08–1.52)	0.005

Complete-case and MICE estimates were directionally consistent.

Supplementary Table S3. Proportional hazards, collinearity, spline, and imputation checks.

Diagnostic or sensitivity check	Result	Interpretation
Troponin T non-linearity	P = 0.186	No evidence of non-linearity.
LVEF non-linearity	P = 0.016	Evidence of non-linearity; interpretation kept continuous and conservative.
Maximum variance inflation factor	2.15	No problematic collinearity detected.
PH diagnostic: Troponin T × LVEF interaction	P = 0.019	Potential time-varying behavior in approximate diagnostic; interpret cautiously.
PH diagnostic: Troponin T, log-transformed and centered	P = 0.165	Approximate residual-time correlation not statistically significant.
PH diagnostic: LVEF, centered percentage points	P = 0.541	Approximate residual-time correlation not statistically significant.
MICE: Troponin T, log-transformed/uncentered	1.12 (1.02–1.23); P = 0.019	Direction consistent with complete-case analysis.
MICE: LVEF/uncentered	0.99 (0.99–1.00); P = 0.030	Direction consistent with complete-case analysis.
MICE: interaction	1.00 (1.00–1.00); P = 0.261	Direction consistent with complete-case analysis.

The interaction term showed possible time-varying behavior and was not used to support mechanistic inference.

Supplementary Table S4. Prespecified subgroup interaction tests.

Subgroup	Exposure	P for interaction
Cardiogenic shock	Troponin T	0.623
Cardiogenic shock	LVEF	0.838
Acute myocardial infarction	Troponin T	0.025
Acute myocardial infarction	LVEF	0.680
Chronic kidney disease	Troponin T	0.046
Chronic kidney disease	LVEF	0.210
Echocardiography after ICU admission	Troponin T	0.698
Echocardiography after ICU admission	LVEF	0.923

No subgroup interaction changed the primary interpretation.

Supplementary Table S5. TRV availability audit.

Analysis set	N	28-day deaths	Proportion	Interpretation
Final ICU HF cohort	4362	1072 (24.6%)	100.0%	All eligible patients with heart failure-coded ICU admissions and 28-day outcome data.
Primary complete-case model	2087	659 (31.6%)	47.8%	Patients with troponin T, numeric LVEF, and complete adjustment covariates.
Measurable-TRV subset	1546	498 (32.2%)	35.4%	Secondary complete-case subset with TRV available.
No measurable TRV in final cohort	2816	574 (20.4%)	64.6%	Patients excluded from TRV-specific modeling.
No measurable TRV within primary complete-case model	541	161 (29.8%)	25.9% of primary complete-case sample	Patients included in primary model but not in TRV subset.

This table was added to clarify the representativeness limitation of the TRV subset.
