# Supplementary material for: Troponin T, Left Ventricular Ejection Fraction, and Tricuspid Regurgitation Velocity for Biomarker- and Echocardiography-Based Risk Stratification in Critically Ill Patients with Heart Failure
Source: Int J Mol Sci. 2026 Jun 13;27(12):5339. doi: 10.3390/ijms27125339 (PMC13299282; doi:10.3390/ijms27125339)
Supplement: Supplementary file 1 [file ijms-27-05339-s001.zip › Figures S1-S5.pdf]

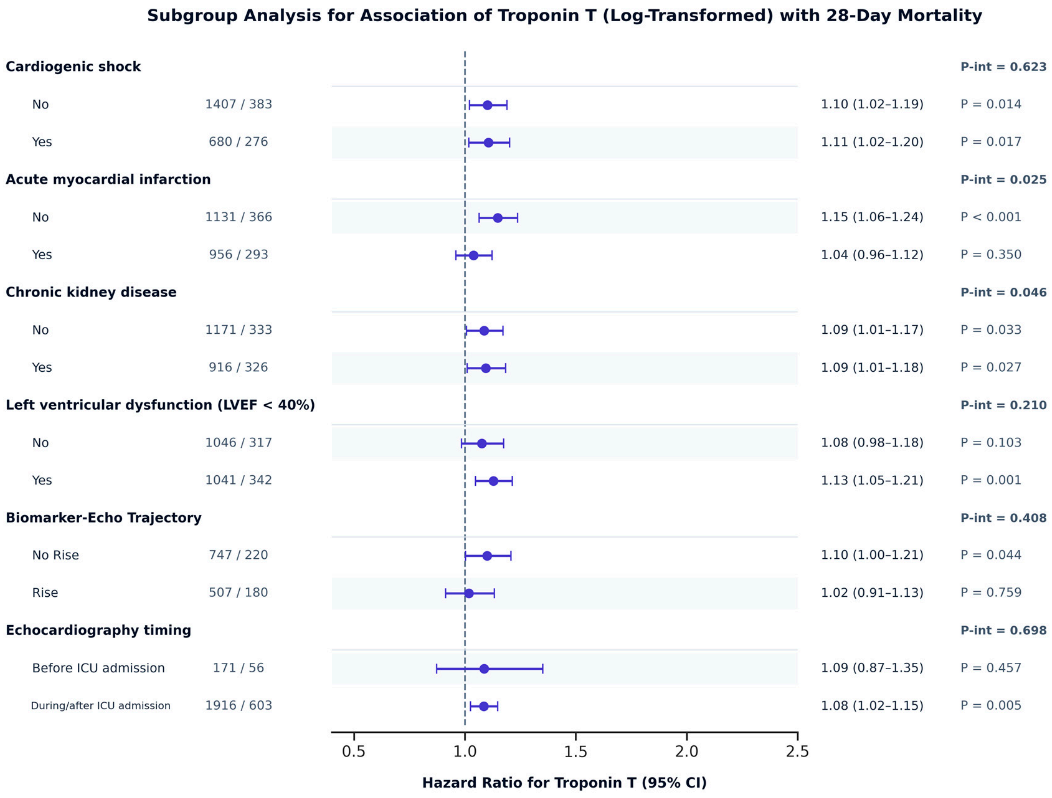

Supplementary Figure S1. Subgroup forest plot for the association of log-transformed troponin T with 28-day mortality.

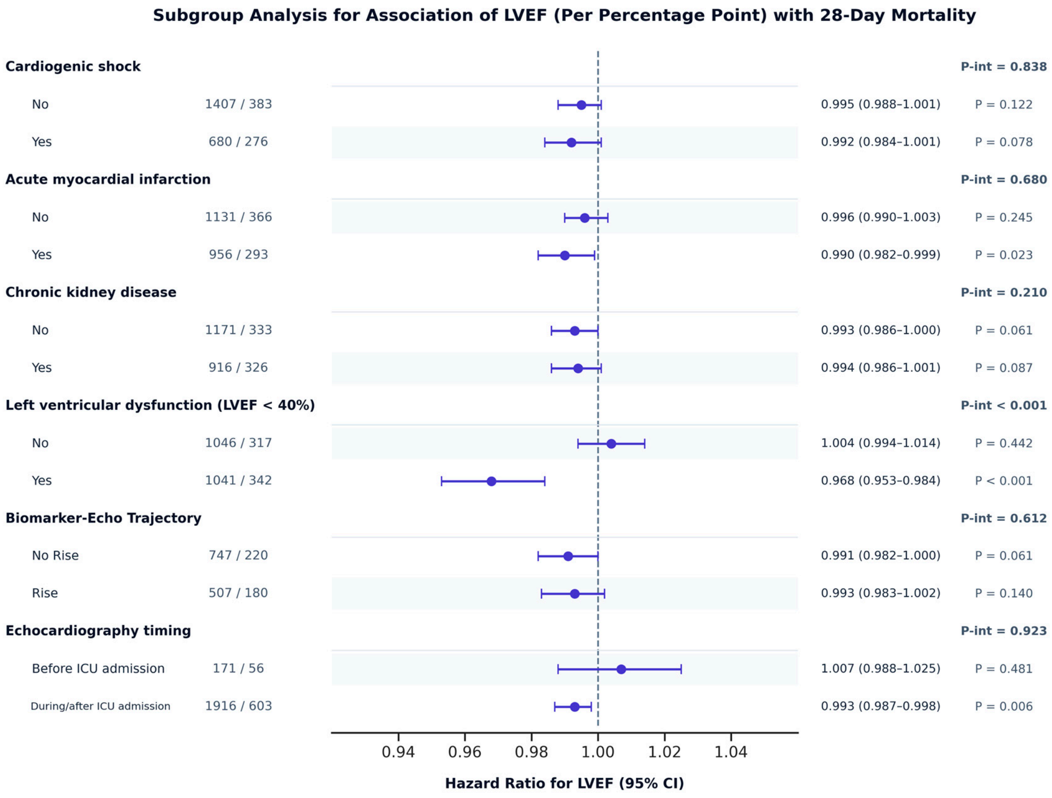

Supplementary Figure S**2.** Subgroup forest plot for the association of LVEF with 28-day mortality.

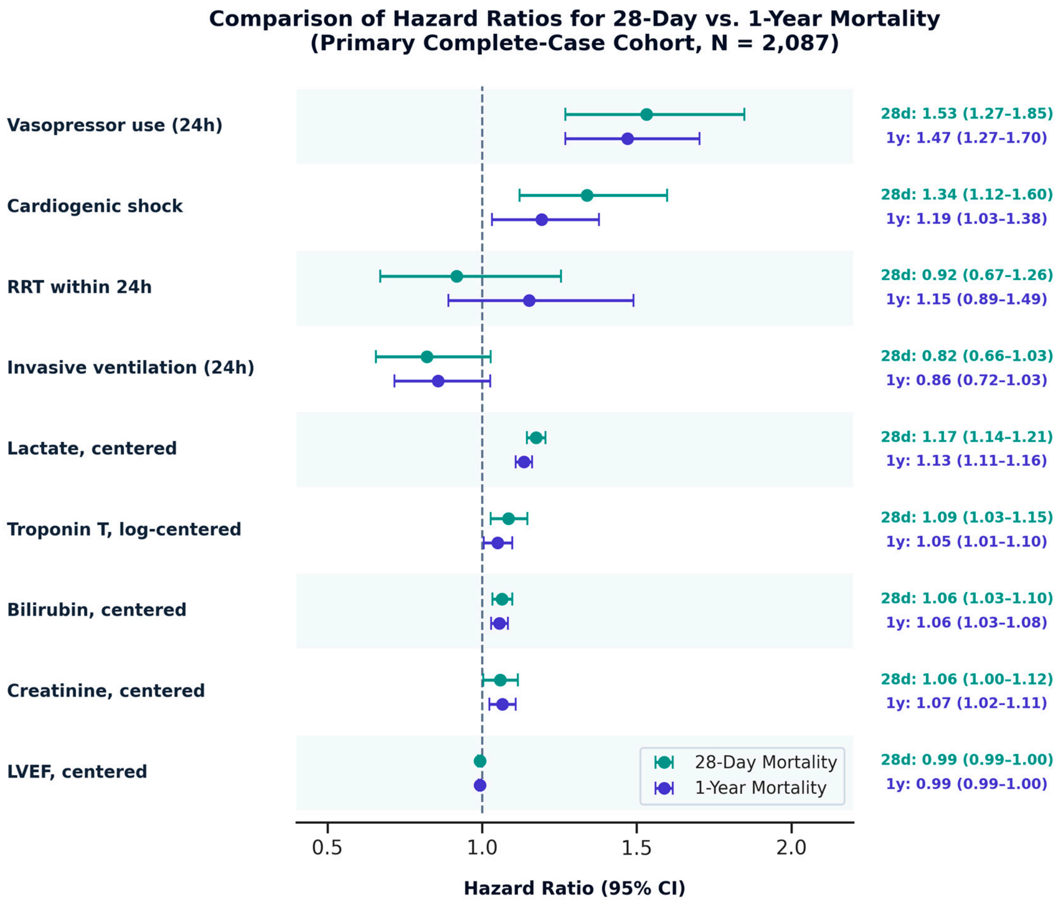

Supplementary Figure S3. Endpoint comparison for 28-day and one-year mortality models.

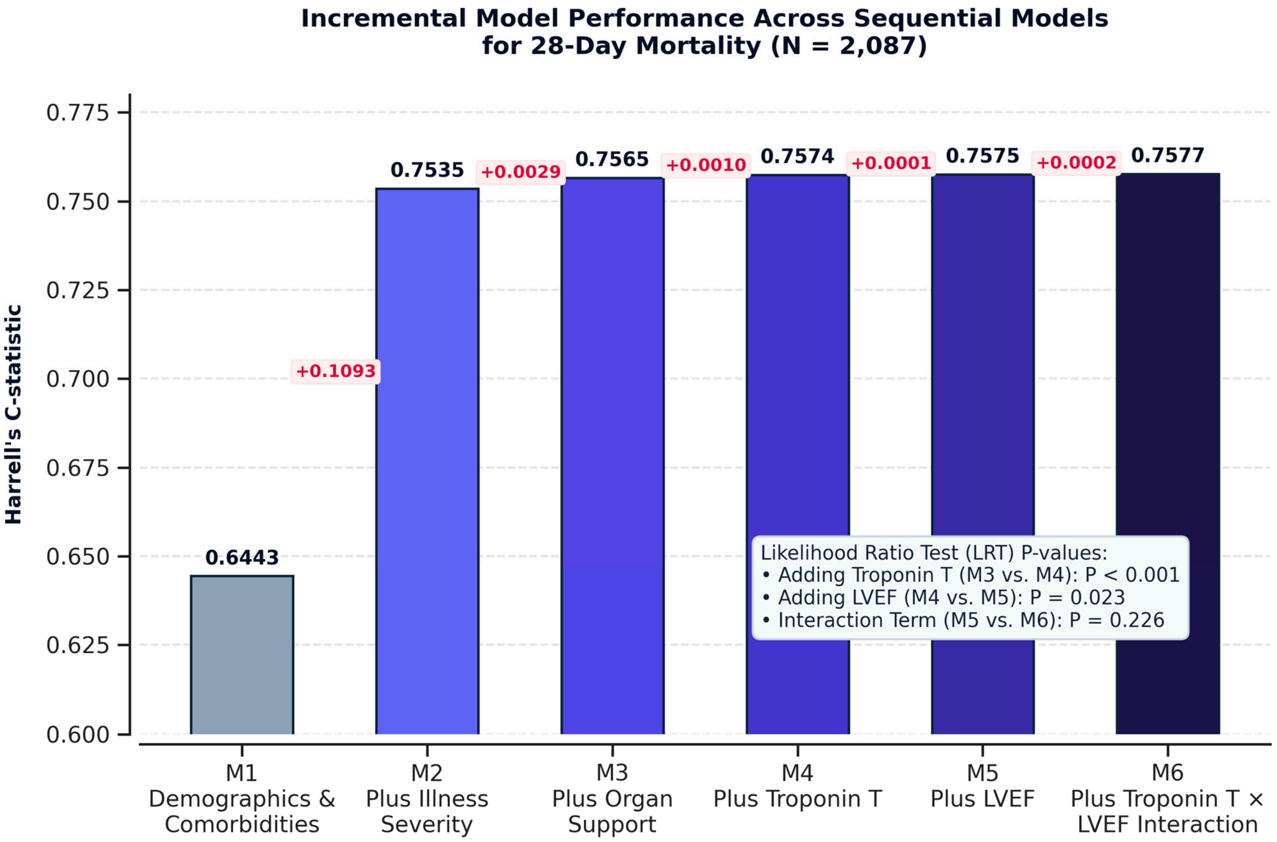

Supplementary Figure S4. Incremental discrimination across sequential models, highlighting the modest C-statistic gains after severity and organ-support variables were entered.

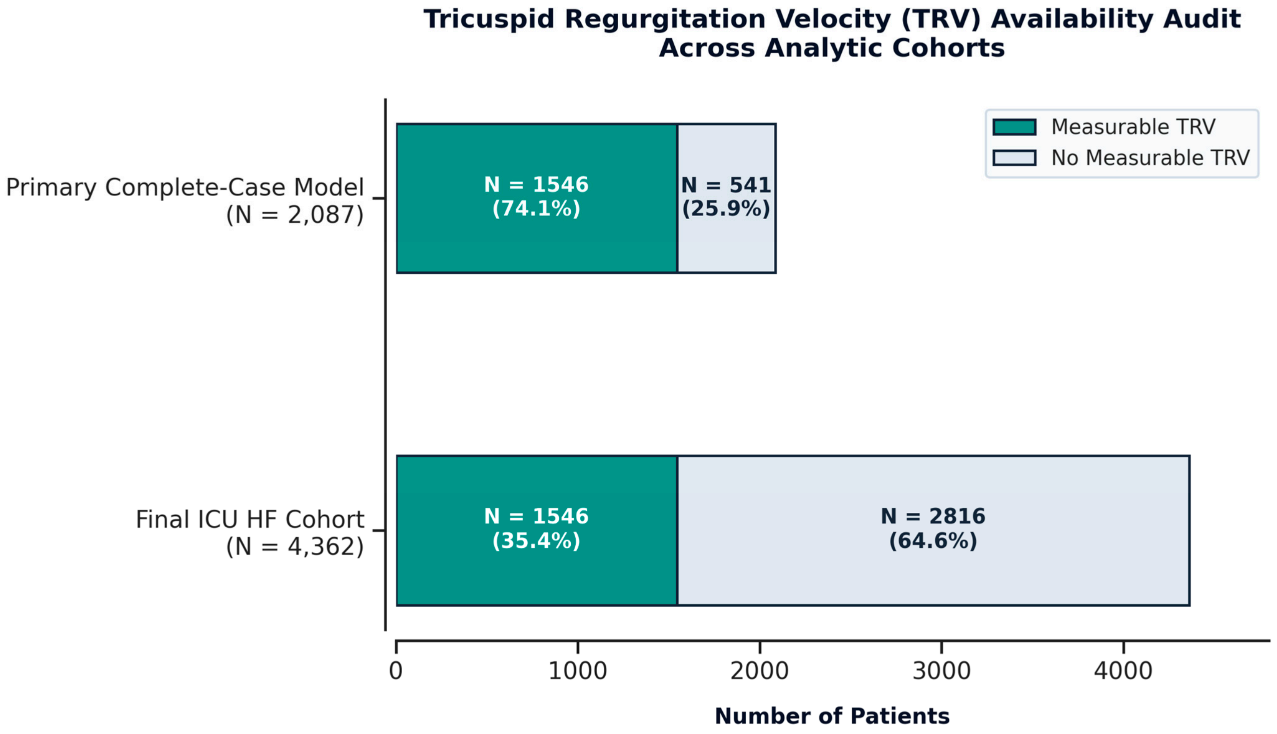

Supplementary Figure S5. TRV availability audit showing the size of the measurable-TRV subset relative to the final cohort and primary complete-case model.
